# A comparative performance analysis of ensemble learning and regularized neural networks in cardiovascular risk prediction

**DOI:** 10.3389/fmedt.2026.1902471

**Published:** 2026-08-06

**Authors:** Golam Md Mohiuddin, Md Shakil Hossain, Md Shohel Sayeed

**Affiliations:** 1 Faculty of Information Science and Technology, Multimedia University, Bukit Beruang, Melaka, Malaysia; 2 Department of Computer Science & Engineering, Manarat International University, Dhaka, Bangladesh; 3 Centre for Intelligent Cloud Computing, CoE for Advanced Cloud, Faculty of Information Science and Technology, Multimedia University, Bukit Beruang, Melaka, Malaysia

**Keywords:** classification, deep learning, generalization, heart disease detection, machine learning, reliability

## Abstract

**Introduction:**

Heart disease classification using small structured clinical datasets requires evaluation procedures that account for sampling variability, model-selection bias, probability calibration, and clinically relevant operating characteristics. This study presents an internally validated comparison of classical classifiers, modern tree ensembles, and regularized artificial neural networks using a cleaned Cleveland heart disease dataset containing 303 observations, 13 predictors, and a binary outcome.

**Methods:**

Continuous variables were standardized, nominal variables were one-hot encoded, and all preprocessing transformations were fitted exclusively within training folds. Model performance was evaluated using five-fold outer cross-validation repeated five times, with inner stratified cross-validation for hyperparameter optimisation. Twelve models were assessed using accuracy, sensitivity, specificity, positive and negative predictive values, F1-score, Matthew's correlation coefficient, ROC-AUC, PR-AUC, Brier score, and calibration measures.

**Results:**

CatBoost achieved the highest mean ROC-AUC of 0.909 ± 0.041 and F1-score of 0.862 ± 0.041, while Logistic Regression achieved a comparable ROC-AUC of 0.906 ± 0.041. Random Forest obtained the highest mean PR-AUC of 0.916 ± 0.040. CatBoost did not significantly outperform Logistic Regression in ROC-AUC, PR-AUC, F1-score, or paired classification errors. Held-out permutation analysis identified the number of major vessels, thalassemia status, and chest-pain category as the most influential predictors.

**Discussion:**

The findings indicate that no model family was universally superior and that a carefully regularized linear model remained competitive with substantially more complex approaches. These findings represent internal validation only and require confirmation using independent clinical cohorts before deployment.

## 1 Introduction

The ability to analyse health-related data and the identification of people who can be affected with heart disease has become one of the main goals of the contemporary healthcare research. Heart disease is a complicated combination of factors such as physiological, demographic, and lifestyle factors and thus detection of heart disease at early stages is always a difficult but crucial task. The development of computational techniques has offered opportunities to assist clinical decisions by the use of automated prediction models that have the capability of processing several risk factors and offering timely risk assessment. Machine learning (ML) has been broadly utilized in the prediction of heart diseases because it is effective in the processing of structured medical data. Traditional ML models, like Logistic Regression, support vector machines, and tree-based ensemble models have proven to be very powerful in binary classification problems, as well as the detection of cardiovascular conditions (1). These are typically used with small data sets due to their efficiency in computation, stability, and are relatively easy to interpret. Specifically, the ensemble-based methods are best applicable in the case of nonlinear relationships between clinical features and strong predictive performance. In parallel, the deep learning (DL) methods have been noted due to their ability to learn complicated feature representations. Artificial neural networks have been used to solve cardiovascular prediction problems, in which it is hoped that a higher-order interaction of risk factors can be better modeled (2). Although DL models have greater representational capability, their performance depends on the size of the datasets, feature design and training stability. Deep architectures can overfit when trained on small and structured data and do not generalize well across data splits.

In spite of the promising outcomes of a great number of studies based on the use of ML and DL models, many of them are concerned with high accuracy without due consideration of reliability and generalization. In medical systems, predictive systems have to exhibit stable performance under different data partition and learning environments. A model that works well in one split of evaluation may not always be stable when presented with novel data, and this is where the issue of the practical applicability of the model in the real-life clinical setting comes in. It is therefore important to test prediction models using various measures and validation schemes in order to have reliable decision support (3). The benefits of early and accurate prediction of heart disease are enormous and include timely correction, decrease in the financial expenses associated with health and enhancement of the quality of life of the patients with risk factors. Predictive models are likely to help clinicians to detect high-risk populations and guide preventative actions based on the specifics of the risk profile (4). Regarding the social health concern, sound prediction systems would be involved in resource distribution and planning of disease management in the long run.

Motivated by these considerations, this paper focuses on evaluating the reliability and generalization of optimized machine learning and deep learning models for heart disease detection. An experimental pipeline is created systematically, which includes standardized preprocessing, class imbalance, stratified cross-validation, and massive hyperparameter optimisation. Various classical ML models are compared to a variety of deep neural network structures to determine the stability of their performance when evaluated based on different metrics of evaluation such as accuracy, F1-score, receiver operating characteristic area under the curve (ROC-AUC), and precision, recall area under the curve. The primary contribution of this work lies in providing a reliability-centred comparison of ML and DL models for heart disease detection using structured cardiovascular data. By emphasizing generalization performance rather than accuracy alone, this study offers practical insights into model selection for predictive healthcare applications. This study does not propose a new classification algorithm. Instead, its contribution is a methodologically controlled comparison of linear, kernel-based, ensemble, boosting, and regularized neural-network approaches under a common internal-validation framework. The revised evaluation incorporates leakage-safe mixed-type preprocessing, repeated nested stratified cross-validation, modern boosting baselines, repeated neural-network initialization, uncertainty estimation, paired statistical comparisons, probability-calibration analysis, class-weight sensitivity analysis, exploratory decision-curve analysis, and held-out permutation importance. The objective is to determine whether observed differences between model families remain consistent after accounting for data-partition variability and model-selection uncertainty on a small structured clinical dataset.

The structure of the paper is as follows: Section 2 reviews related work on heart disease prediction using ML and DL methods. Section 3 describes the dataset and methodology employed in this study. Section 4 presents experimental results and comparative analysis. Section 5 concludes the paper and outlines directions for future research.

## 2 Literature review

Prediction of heart disease has been a dynamic field of research in the ML community with many studies examining how data-driven models could be used to aid in the early detection and risk mechanism. Researchers from both academic and applied domains have investigated a wide range of algorithms to analyse cardiovascular data and identify patients at elevated risk of heart-related conditions. The initial research in this area was mainly centred on classical ML methods because of their computational efficiency and their ability to be applied to structured medical data. Logistic Regression (LR), Naive Bayes (NB), Decision Trees (DT), Support Vector Machines (SVM), and k-Nearest Neighbors (KNN) are among the many algorithms that have been researched in regard to the classification of heart diseases (5). Hybrid machine learning model that combines feature selection and multiple classifiers and showed a higher prediction accuracy than models based on one model (6). The main findings of their study are that the use of complementary learning strategies is essential in predictive performance in cardiovascular risk assessment.

Various comparative works have been carried out comparing different ML models on the same dataset to ascertain their comparative merits. The prediction of heart attacks based on the classification of Naive Bayes and Decision Tree and found that Decision Trees always performs better than Bayesian classifiers given an equal dataset of clinical conditions (7). This observation indicates that tree models should be better used to capture nonlinear relationships between cardiovascular risk factors. On the same note, Kohsasih et al. (8) used Decision Tree and Naive Bayes models on a public dataset on heart disease and revealed that the proper pre-processing and handling of missing values made the model more reliable, with better results using Decision Trees. Due to progress in computational power, there has been an emergence of deep learning methods applied to predicting heart disease. Complex interactions between clinical attributes have been modeled using artificial neural networks multilayer perceptron's. A deep learning-based framework propose with neural network architectures to identify the risk of heart disease was higher in accuracy than some of the traditional ML models (9). These researches indicate that deep learning is potentially beneficial provided that adequate data and suitable regularization mechanisms are used.

Subsequent researches have been used to compare deep learning models and traditional classifiers to determine their relative efficacy. ANN-based classifiers with Decision Trees and Naive Bayes models and discovered that neural networks were more accurate in predictions in some cases (10). They also focused on the impact of lifestyle-related factors, including smoking and obesity, on the results of the prediction in their work. However, deep learning models are sensitive to tuning and can have unstable performance when they are trained on small sets. Other scholars have concentrated on maximizing computational efficiency as well as minimizing model complexity. Bayes Net and Sequential Minimal Optimisation (SMO) classifiers and proved that the cost of the computational process could be reduced by the dimensionality reduction methods without decreasing the rate of classification (11). Their research used several measures to perform performance evaluation such as prediction accuracy and ROC-AUC, which supports the importance of multi-metric evaluation in medical prediction problems.

Although the prediction of heart disease has received a lot of literature, there are still a number of limitations to literature. Most studies make use of a single train-test split and this limits evaluation of model generalization. Furthermore, accuracy based comparisons dominate performance evaluation which does not necessarily indicate model reliability especially when there is class imbalance in the dataset. Deep learning models are often used without adequate validation and regularization, which increases the likelihood of overfitting and unreliable generalization (12). In current studies, the focus has been on the role of reliability and generalization in healthcare prediction systems. Strong evaluation techniques, including stratified cross-validation, class-balanced learning, and hyperparameter optimisation are becoming considered the keys to the stability of model behaviour in unseen data (13). Some studies highlight both the promise and limitations of ML for heart disease diagnosis. Sharma et al. (14) identified key gaps in validation, dataset diversity, interpretability, and external testing. Reddy et al. (15) and Shah et al. (16) compared multiple classifiers using the Cleveland dataset and demonstrated that performance depends strongly on model selection and validation strategy. Elshewey et al. (17) further showed that optimisation-enhanced LSTM models can achieve strong results. These findings support the need for rigorous cross-validation, statistical testing, explainability, calibration, and external validation.

Nevertheless, there is little in the way of thorough studies that comparatively contrast optimized machine learning and deep learning models within a single evaluation framework. This gap is filled by the current research that provides a reliability-focused comparison of the conventional machine learning and deep learning models in detecting heart disease. Through stratified k-fold cross-validation, equal definition of weights of classes, as well as extensive hyperparameter tuning, this work entails a comprehensive comparison of model stability and generalization. In contrast to the previous research, which pays much attention to accuracy, the present study will pay attention to the predictive behaviour consistency across different evaluation metrics, which will offer practical information to the implementation of predictive models into clinical decision-support systems.

## 3 Proposed methodology

**Figure 1:**
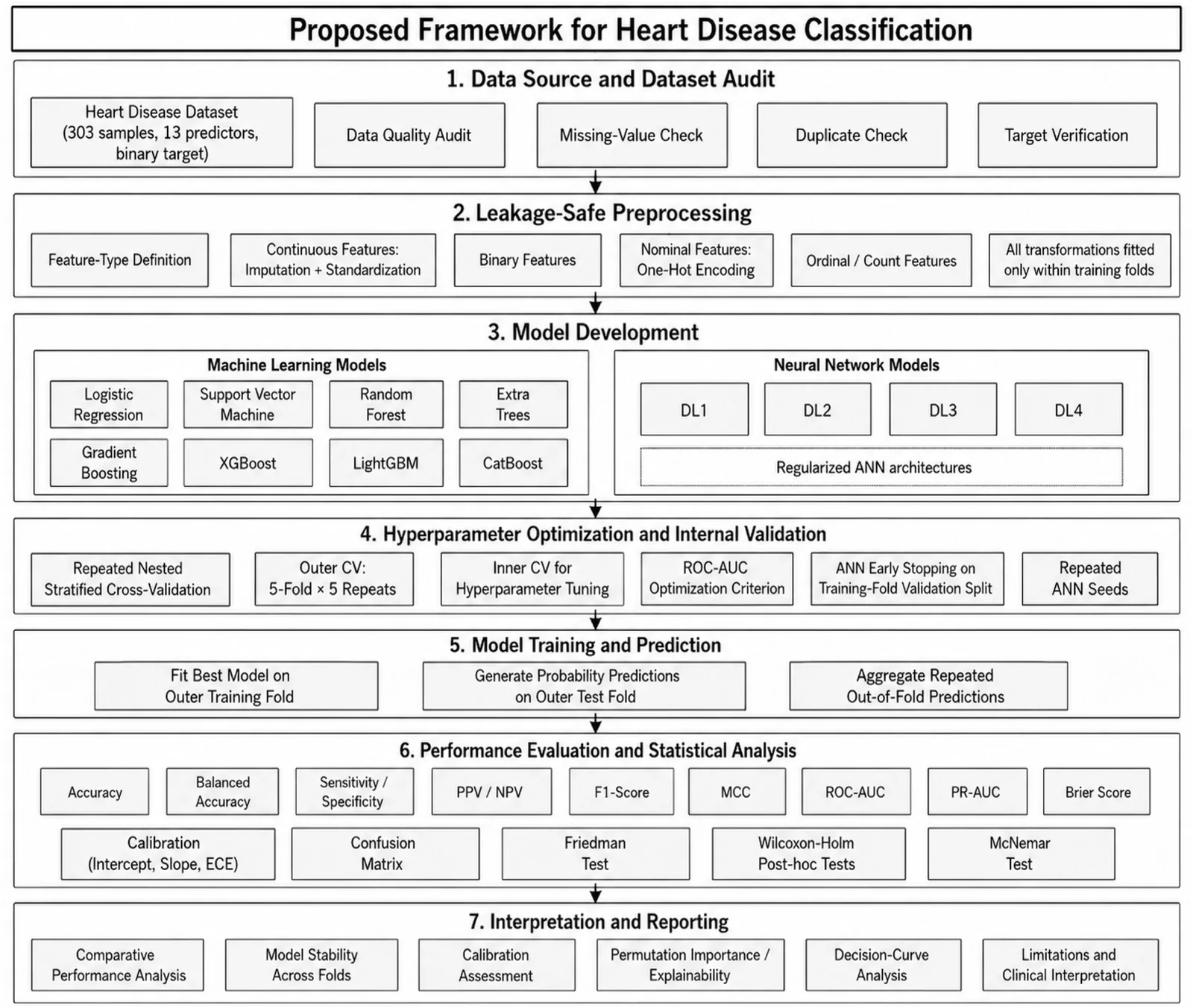
Proposed framework for heart disease prediction.

This section presents the experimental framework adopted to evaluate the reliability and generalization capability of optimized machine learning and deep learning models for heart disease detection. The methodology consists of dataset analysis, preprocessing, model development, hyperparameter optimisation, and multi-metric performance evaluation under a controlled and reproducible setting. The overall workflow of the proposed heart disease prediction framework is illustrated in Figure 1.

### 3.1 Dataset description

This study was conducted using the Cleveland subset of the Heart Disease dataset obtained from the UCI Machine Learning Repository (18). The analysed file contained 303 observations, 13 predictor variables, and one binary target variable. The target was already encoded as 0 for absence and 1 for presence of heart disease; therefore, no additional conversion from the original UCI diagnostic scale of 0–4 was required. The final dataset contained 165 positive observations, corresponding to 54.46%, and 138 negative observations, corresponding to 45.54%.

An explicit data-quality audit identified no missing values in the analysed file. This differs from the raw Cleveland dataset, which is commonly reported to contain missing entries in variables such as ca and thal, and confirms that the present analysis used a cleaned derivative rather than the unprocessed UCI file. Imputation steps were nevertheless retained inside the modelling pipelines to ensure methodological robustness and compatibility with future datasets.

One pair of observations was identical across all predictors and the outcome. Because patient identifiers were unavailable, it was not possible to determine whether these represented a duplicated observation or two patients with identical recorded characteristics. A sensitivity analysis excluding one copy produced nearly unchanged performance for the leading models. Therefore, all 303 benchmark observations were retained in the primary analysis, and this issue is acknowledged as a limitation.

### 3.2 Data pre-processing

To ensure methodological consistency and prevent data leakage, a structured preprocessing pipeline was applied uniformly across all models. The dataset was first divided into input features *X* and corresponding target labels *y*, followed by an 80–20 train test split using stratified sampling to preserve the original class distribution in both subsets. This approach maintains representativeness in the evaluation phase and reduces sampling bias. Although the dataset did not contain substantial missing values, median imputation was incorporated within the pipeline to enhance robustness and reproducibility. All numerical features were standardized using z-score normalization as defined in Equation 1, computed as

$$x^{\prime }=(x-\mu )/\sigma$$ (1)

where μ and σ denote the mean and standard deviation calculated from the training data. Standardisation was particularly important for algorithms sensitive to feature magnitude, such as Logistic Regression and Support Vector Machines, as it ensures stable convergence during optimisation. Additionally, balanced class weights were introduced during model training to account for the slight class disproportion present in the dataset. By penalizing misclassification of minority samples more heavily, this strategy promotes balanced decision boundaries and supports improved generalization performance.

### 3.3 Model selection

Eight ML models were evaluated: Logistic Regression, Support Vector Machine, Random Forest, Extra Trees, Gradient Boosting, XGBoost, LightGBM, and CatBoost. Logistic Regression served as an interpretable linear baseline, while Support Vector Machine represented margin-based classification. Random Forest and Extra Trees represented randomized bagging ensembles. Gradient Boosting, XGBoost, LightGBM, and CatBoost were included to compare conventional boosting with recent optimized implementations. All classifiers received identically processed data and were evaluated using the same outer test folds.

#### 3.3.1 Machine learning models

Four widely adopted supervised classification algorithms were selected based on their established performance in medical prediction tasks:

- Logistic Regression (LR): Logistic Regression was used as a baseline linear classifier because of its simplicity, interpretability, and strong performance in binary classification problems. In medical prediction tasks, interpretability is important because the relationship between input risk factors and the predicted outcome should be understandable. Logistic Regression estimates the probability of heart disease by modelling the relationship between independent clinical features and the binary target variable. Since it produces probabilistic outputs, it is also suitable for threshold-independent evaluation using ROC-AUC. Although LR assumes a linear relationship between predictors and outcome, it provides a useful reference point for comparing more complex nonlinear models.

Logistic Regression estimates the probability that a patient belongs to the heart disease class using the sigmoid function as shown in Equation 2:

$$P(y=1|x)=\sigma (\omega^{T}x+b)=\frac{1}{1+e^{-(\omega^{T}x+b)}}$$ (2)

Here, *x* represents the input clinical features, ω represents the feature weights, and *b* is the bias term. The model converts a weighted linear combination of cardiovascular risk features into a probability score for binary classification (19).

- Random Forest (RF): Random Forest was selected as an ensemble-based learning method that combines multiple decision trees to improve prediction stability and reduce model variance. Each tree in the forest is trained on a different subset of the data, and the final prediction is obtained through majority voting. This makes Random Forest effective in capturing nonlinear relationships and complex interactions among cardiovascular risk factors such as age, chest pain type, cholesterol level, maximum heart rate, and ST depression. Another advantage of RF is its robustness against overfitting compared with a single decision tree. In addition, Random Forest can estimate feature importance, which is useful for identifying influential clinical variables contributing to heart disease prediction.

Random Forest combines multiple decision trees and produces the final class prediction using majority voting as shown in Equation 3:

$$\hat{y}=mode\{T_{1}(x),T_{2}(x),\ldots ,T_{B}(x)\}$$ (3)

where *T_B* (*x*) denotes the prediction of the B decision tree. In this study, Random Forest is useful because it can capture nonlinear relationships among clinical variables while reducing the variance of a single decision tree. Breiman introduced Random Forests as ensembles of tree predictors, where each tree depends on randomly sampled variables (20).

- Support Vector Machine (SVM): It was included because of its strong theoretical foundation and effectiveness in high-dimensional classification problems. SVM works by identifying an optimal hyperplane that maximizes the margin between different classes. A larger margin generally improves the model's ability to generalize to unseen data. In this study, both linear and radial basis function kernels were considered to examine whether the dataset follows a linear or nonlinear decision boundary. The linear kernel provides a simpler and more interpretable separation, while the RBF kernel allows the model to capture more complex nonlinear patterns among clinical features.

Support Vector Machine aims to identify the optimal separating hyperplane by maximizing the margin between two classes. Its soft-margin optimisation objective is given in Equation 4:

$$\underset{w,b\xi }{\min}\frac{1}{2}|\omega^{2}|+C\overset{n}{\underset{i=1}{\sum }}\xi_{i}$$ (4)

where *C* controls the trade-off between margin maximization and classification error. In heart disease prediction, SVM helps separate positive and negative cases by maximizing the decision boundary margin. Cortes and Vapnik introduced Support Vector Machines (SVMs) as a supervised learning method for binary classification by maximizing the margin between classes, providing strong generalization performance in high-dimensional spaces (21).

- Gradient Boosting (GB): It was used as a boosting-based ensemble model that builds trees sequentially, where each new tree attempts to correct the errors made by the previous trees. Unlike Random Forest, which builds trees independently, Gradient Boosting improves performance through an iterative learning process. This allows the model to gradually reduce classification error and capture subtle patterns in the dataset. GB is particularly useful for structured tabular data because it can model nonlinear relationships and feature interactions effectively. However, since boosting models can be sensitive to hyperparameter settings, careful tuning was applied to control model complexity and improve generalization performance.

Gradient Boosting builds an additive model by sequentially adding weak learners to correct previous prediction errors as shown in Equation 5:

$$F_{M}(x)=F_{0}(x)+\overset{M}{\underset{m=1}{\sum }}vh_{m}(x)\;$$ (5)

where *F_M* (*x*) is the final boosted model, *h_m* is the weak learner added at iteration m, and v is the learning rate. This method improves predictive performance by gradually minimizing the loss function through sequential model refinement. Friedman introduced Gradient Boosting as a greedy function approximation method that sequentially builds weak learners to minimize a specified loss function and improve predictive performance (22).

- Extra Trees (ET): Extra Trees (Extremely Randomized Trees) was selected as an ensemble-based classifier that introduces additional randomness during the tree construction process. Unlike Random Forest, which selects the optimal split among a random subset of features, Extra Trees randomly selects both the feature and the split threshold at each node. This increased randomization helps reduce model variance and computational complexity while improving generalization performance. Extra Trees is particularly effective for structured clinical datasets because it can capture nonlinear relationships among cardiovascular risk factors without extensive preprocessing. Moreover, its ensemble mechanism makes it robust to noisy data and less prone to overfitting.

The final prediction of Extra Trees is obtained through majority voting among all randomized decision trees as shown in Equation 6:

$$\hat{y}=mode\{T_{1}(x),T_{2}(x),\ldots ,T_{N}(x)\}$$ (6)

where *T_N_ (x)* denotes the prediction of the *N^th^* randomized decision tree. The additional randomness introduced during split selection improves model diversity and contributes to better generalization performance on unseen cardiovascular data. Geurts et al. introduced Extremely Randomized Trees as an efficient ensemble learning technique that reduces variance while maintaining predictive accuracy (23).

- Extreme Gradient Boosting (XGBoost): Extreme Gradient Boosting (XGBoost) was included because of its high predictive performance and efficient implementation of Gradient Boosting algorithms. XGBoost incorporates regularization techniques, parallel processing, and optimized tree pruning mechanisms that improve both accuracy and computational efficiency. It is particularly suitable for cardiovascular risk prediction because it can effectively capture complex nonlinear interactions among clinical variables while reducing overfitting. Furthermore, XGBoost supports built-in handling of missing values and provides feature importance measures that enhance model interpretability in healthcare applications.

The objective function of XGBoost is defined in Equation 7:

$$L(t)=\;\overset{n}{\underset{i=1}{\sum }}l(y_{i},{\hat{y}}_{i}(t-1)+ft(xi))+\;\Omega (\;ft)$$ (7)

where *l(⋅)* denotes the loss function, *f_t_* (*x_i_*) represents the newly added tree at iteration t, and *Ω*(*f_t_)* is the regularization term controlling model complexity. The inclusion of regularization enables XGBoost to achieve high predictive performance while maintaining strong generalization capabilities. Chen and Guestrin introduced XGBoost as a scalable and regularized tree boosting framework for machine learning applications (24).

- Light Gradient Boosting Machine (LightGBM): Light Gradient Boosting Machine (LightGBM) was selected because of its computational efficiency and ability to handle large-scale tabular datasets. LightGBM employs a histogram-based learning algorithm and a leaf-wise tree growth strategy, which significantly accelerate training while improving predictive performance. The leaf-wise splitting mechanism allows the model to reduce loss more effectively than conventional level-wise tree growth approaches. In cardiovascular risk prediction, LightGBM is advantageous for capturing complex feature interactions while maintaining efficient model training and low memory consumption.

LightGBM minimizes the objective function by iteratively optimizing the loss function and regularization term as shown in Equation 8:

$$Objective=\overset{n}{\underset{i=1}{\sum }}l(y_{i},{\hat{y}}_{i})+\overset{k}{\underset{k=1}{\sum }}\Omega (Fk)\;$$ (8)

where *l (_⋅_)* represents the prediction loss, *f_k_* denotes the decision tree model, and *Ω(f_k_)* controls model complexity through regularization. The histogram-based optimisation and leaf-wise splitting strategy contribute to its superior computational efficiency and predictive capability. Ke et al. introduced LightGBM as a highly efficient Gradient Boosting decision tree framework (25).

- Categorical Boosting (CatBoost): CatBoost was included because of its capability to reduce prediction bias and efficiently process categorical features. Although the heart disease dataset primarily consists of numerical variables, several clinical attributes such as chest pain type, resting electrocardiographic results, and thalassemia categories can benefit from CatBoost's specialized encoding strategy. Unlike conventional boosting algorithms, CatBoost employs ordered boosting techniques that mitigate target leakage and reduce overfitting during training. Its robust handling of categorical information and strong generalization performance make it particularly suitable for healthcare prediction tasks.

CatBoost builds an additive ensemble model using Gradient Boosting and can be expressed as shown in Equation 9:

$$F(x)=\overset{M}{\underset{m=1}{\sum }}\alpha m\;hm(x)$$ (9)

where *h_m_ (x)* denotes the weak learner at iteration *m,* and *α_m_* represents its corresponding weight. CatBoost utilizes ordered boosting and target statistics to improve prediction stability and minimize overfitting in classification problems. Prokhorenkova et al. introduced CatBoost as an unbiased Gradient Boosting algorithm with effective categorical feature handling (26). These models were embedded within preprocessing pipelines and subjected to hyperparameter optimisation to ensure fair and unbiased comparison.

#### 3.3.2 Deep learning models

To evaluate whether increased representational capacity improves performance on structured medical data, four Artificial Neural Network (ANN) architectures were implemented with progressively increasing complexity:

- DL1 (Basic ANN)
- DL2 (Deep ANN)
- DL3 (ANN with Dropout)
- DL4 (ANN with Batch Normalization and Dropout)

**Figure 2:**
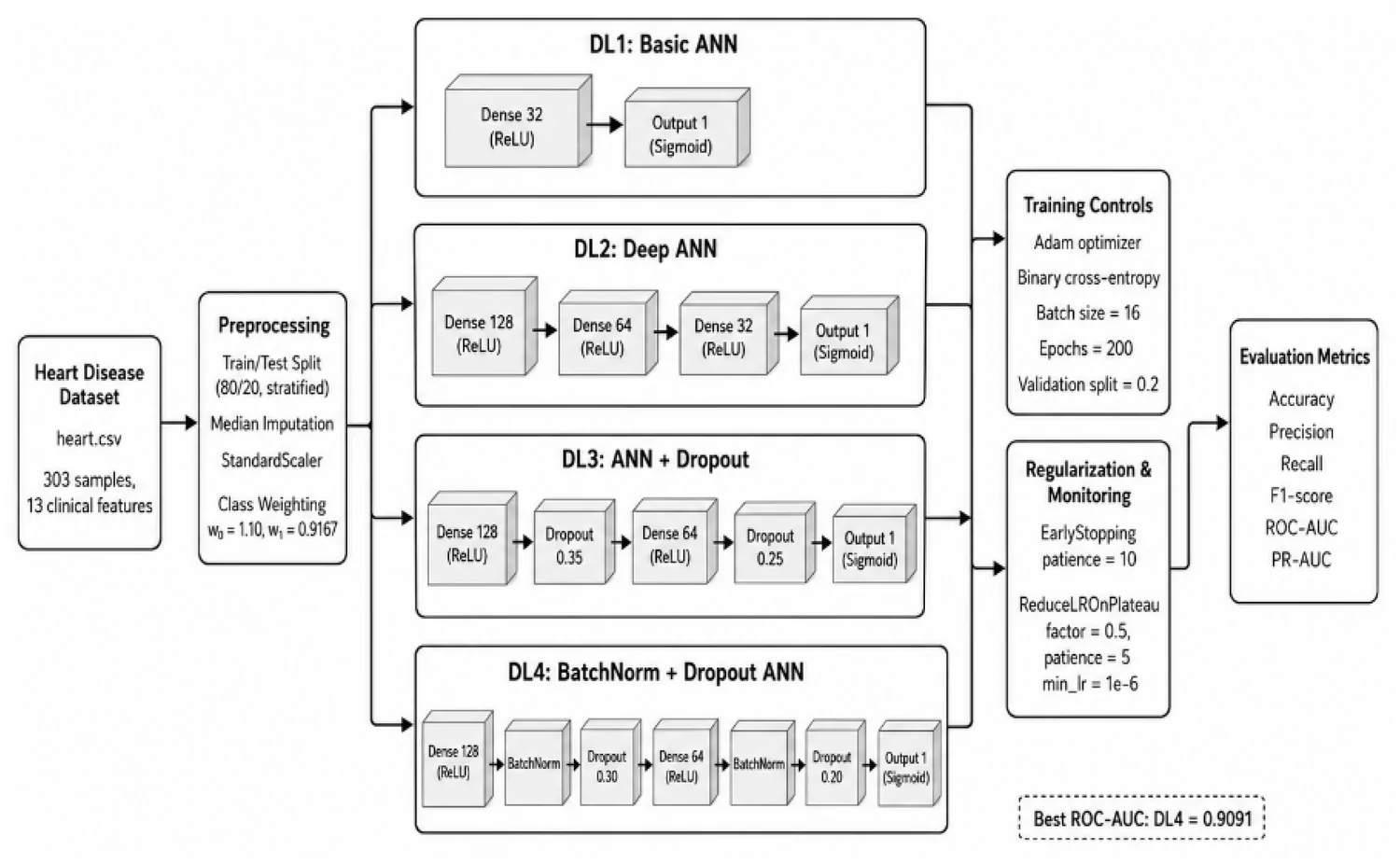
Deep learning algorithm architectures for heart disease prediction.

The framework in Figure 2. illustrates the preprocessing pipeline and four ANN-based models, including a basic ANN, deep ANN, dropout-regularized ANN, and batch normalization with dropout ANN. Each model is trained using class-weighted learning and evaluated using accuracy, precision, recall, F1-score, ROC–AUC, and PR–AUC.

All hidden layers used ReLU activation and He initialization, while the output layer used a single sigmoid unit. The networks were optimized using Adam and binary cross-entropy loss. Table 1 summarizes the architectural configurations of the training used a batch size of 16, a prespecified maximum number of epochs, and early stopping based exclusively on a validation subset of the outer-training data. Each ANN architecture was repeated using multiple random initializations, and seed-specific probabilities were averaged before fold-level evaluation. The outer test folds remained entirely unseen during architecture fitting, early stopping and model selection.

**Table 1:** Architectural configuration of the proposed deep learning models.

Model	Architecture	Dropout	Batch normalization	Trainable parameters
DL1	Input–32–1	None	No	737
DL2	Input–128–64–32–1	None	No	13,185
DL3	Input–128–64–1	0.35 and 0.25	No	11,137
DL4	Input–128–64–1	0.30 and 0.20	After each hidden layer	11,521

### 3.4 Hyperparameter optimisation and cross-validation

Model performance was estimated using repeated nested stratified cross-validation. The outer evaluation consisted of five stratified folds repeated five times, producing 25 held-out outer test evaluations for each model. Identical outer folds were used for all model families. Within each outer-training fold, stratified inner cross-validation was used exclusively for hyperparameter optimisation, with ROC-AUC selected as the optimisation criterion. For each outer fold, the selected model configuration was refitted using the corresponding complete outer-training set and evaluated once on the untouched outer test fold. Preprocessing transformations were included within the inner optimisation pipeline, ensuring that imputation, encoding, and scaling parameters were not estimated using validation or outer-test observations.

Neural-network architectures were prespecified. Early stopping and training-duration selection were conducted using only a validation subset derived from each outer-training fold. Results are reported as mean ± standard deviation across the aligned outer test folds. Repeated out-of-fold probabilities were additionally aggregated at the observation level for confidence intervals, calibration analysis, paired classification-error testing, and exploratory decision-curve analysis.

### 3.5 Evaluation framework

Heart disease presence was defined as the positive class. Predicted probabilities were converted into class labels using the prespecified threshold of 0.50. Performance was evaluated using accuracy, balanced accuracy, sensitivity, specificity, positive predictive value, negative predictive value, F1-score, Matthews correlation coefficient, ROC-AUC, PR-AUC, Brier score, log loss, expected calibration error, calibration intercept, and calibration slope. Results are reported as mean ± standard deviation across the 25 outer test-fold evaluations. Stratified bootstrap confidence intervals were calculated using aggregated repeated out-of-fold probabilities. Overall model differences were examined using the Friedman test. When the Friedman test was significant, paired Wilcoxon signed-rank tests with Holm correction were performed for *post-hoc* comparisons. McNemar's exact test was used to compare paired classification errors between the two leading models. The effect of class weighting was evaluated through a weighted-versus-unweighted sensitivity analysis.

Calibration curves were generated to compare predicted probabilities with observed outcome frequencies. Exploratory decision-curve analysis was performed to estimate net benefit over a range of threshold probabilities. Model interpretation was assessed using permutation importance calculated on held-out outer test folds, thereby avoiding the optimistic importance estimates that can occur when explanations are generated from training data.

## 4 Result and discussion

**Figure 3:**
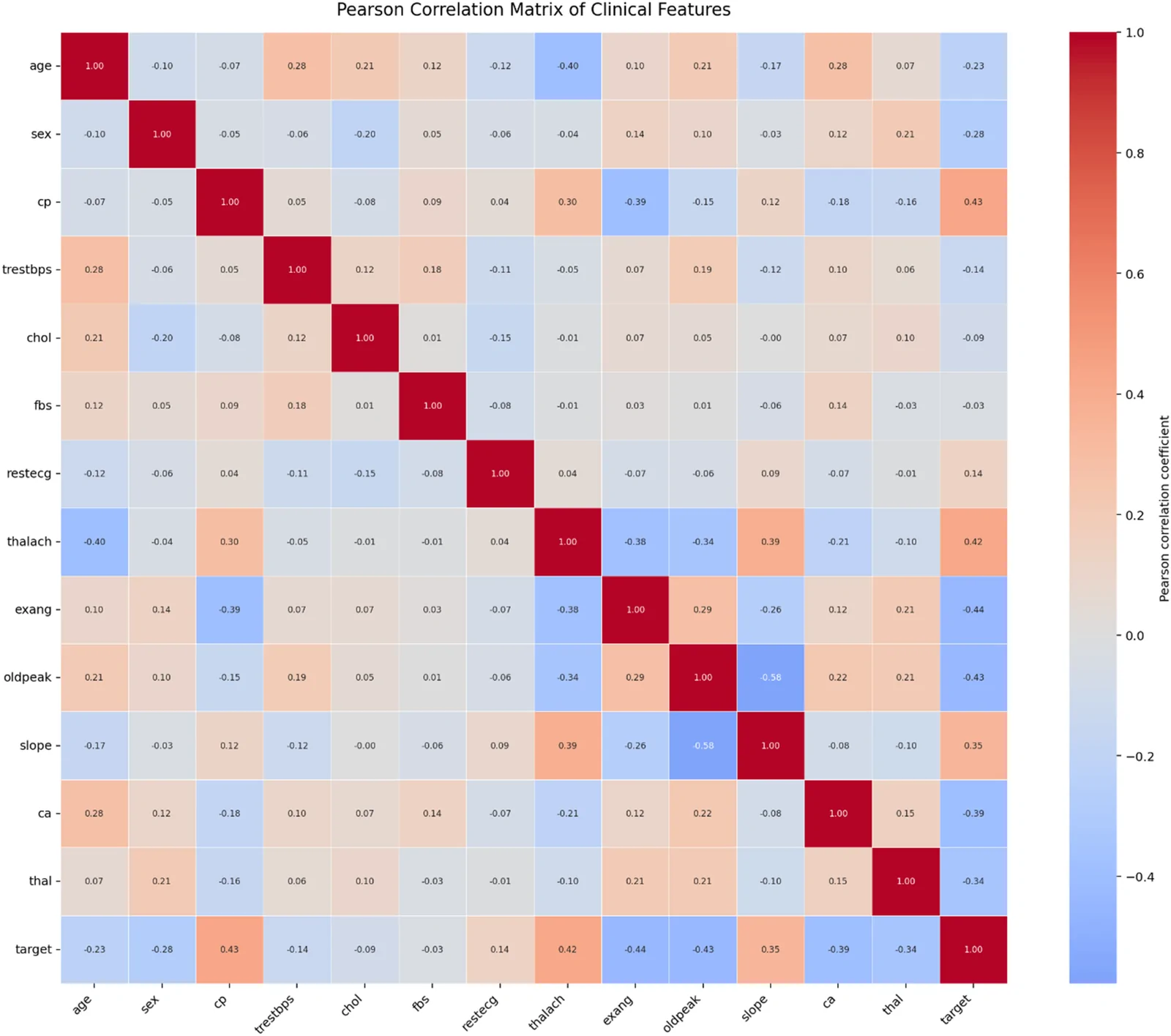
Pearson correlation matrix of the clinical features and target variable.

The experimental analysis included exploratory visualization, repeated model evaluation, statistical comparison, calibration assessment, and model interpretation. Figure 3 presents the complete pearson correlation matrix for the clinical variables and binary target. The corresponding numerical correlation coefficient is displayed inside every cell to two decimal places, while warmer and cooler colors indicate positive and negative associations, respectively. The strongest positive correlations with the target were observed for chest pain category (cp,r=0.43), maximum heart rate (thalach,r=0.42), and ST-segment slope (slope, *r* = 0.35). Exercise-induced angina (exang,r=−0.44), ST depression (oldpeak,r=−0.43), number of major vessels (ca,r=−0.39), and thalassemia status (thal,r=−0.34) showed notable negative correlations with the target. Because several variables are integer-coded categorical or ordinal features, these Pearson coefficients are interpreted descriptively rather than causally.

**Figure 4:**
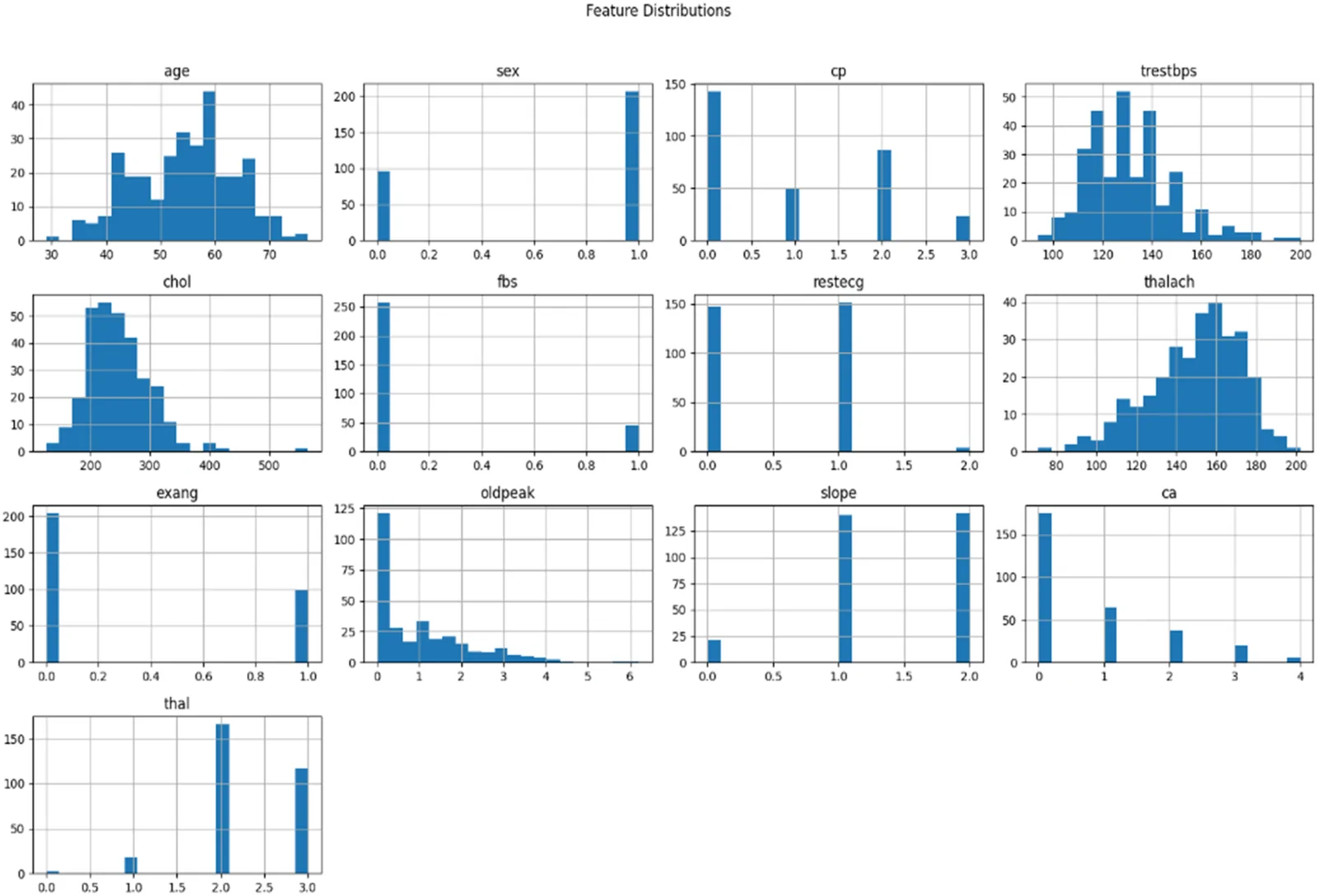
Feature distribution histograms.

Figure 3 presents the complete pearson correlation matrix, with the corresponding numerical coefficient displayed in each cell to improve readability and transparency. Histograms were plotted for all numerical attributes to observe distribution patterns. Figure 4, the graphical analysis shows that heart disease prevalence increases in middle-aged and older individuals. Age distribution indicates that individuals between 50 and 70 years demonstrate higher frequency of positive heart disease cases. Similarly, exercise-induced angina (exang) and ST depression (oldpeak) exhibit visible variation between positive and negative cases, supporting their predictive relevance.

**Figure 5:**
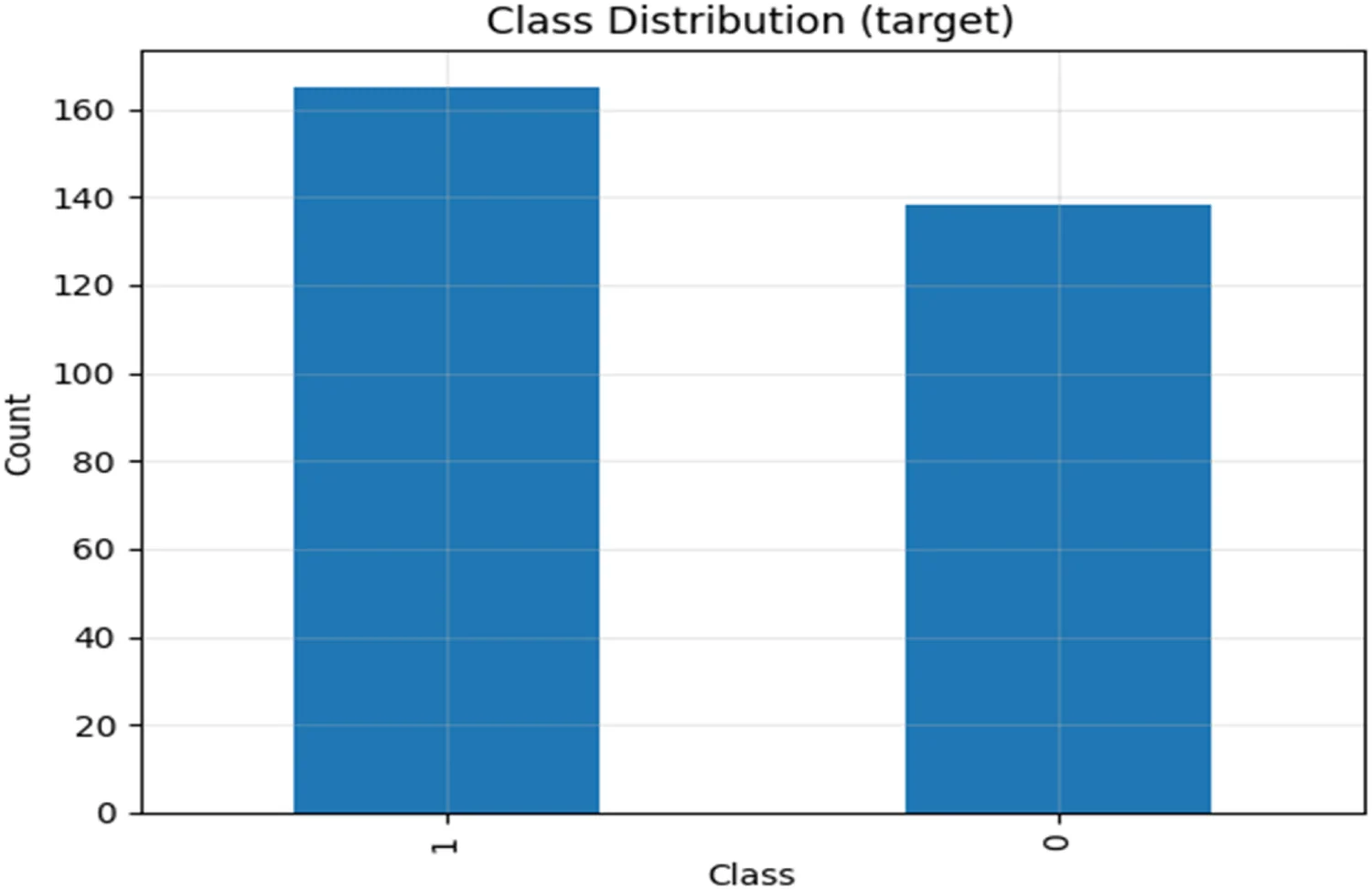
Target class distribution.

A bar graph was created to illustrate class distribution of the target variable in Figure 5. The dataset contains 165 positive cases and 138 negative cases, reflecting a moderately balanced dataset. Although severe imbalance was not observed, class-weight adjustments were applied during training to maintain prediction fairness.

**Figure 6:**
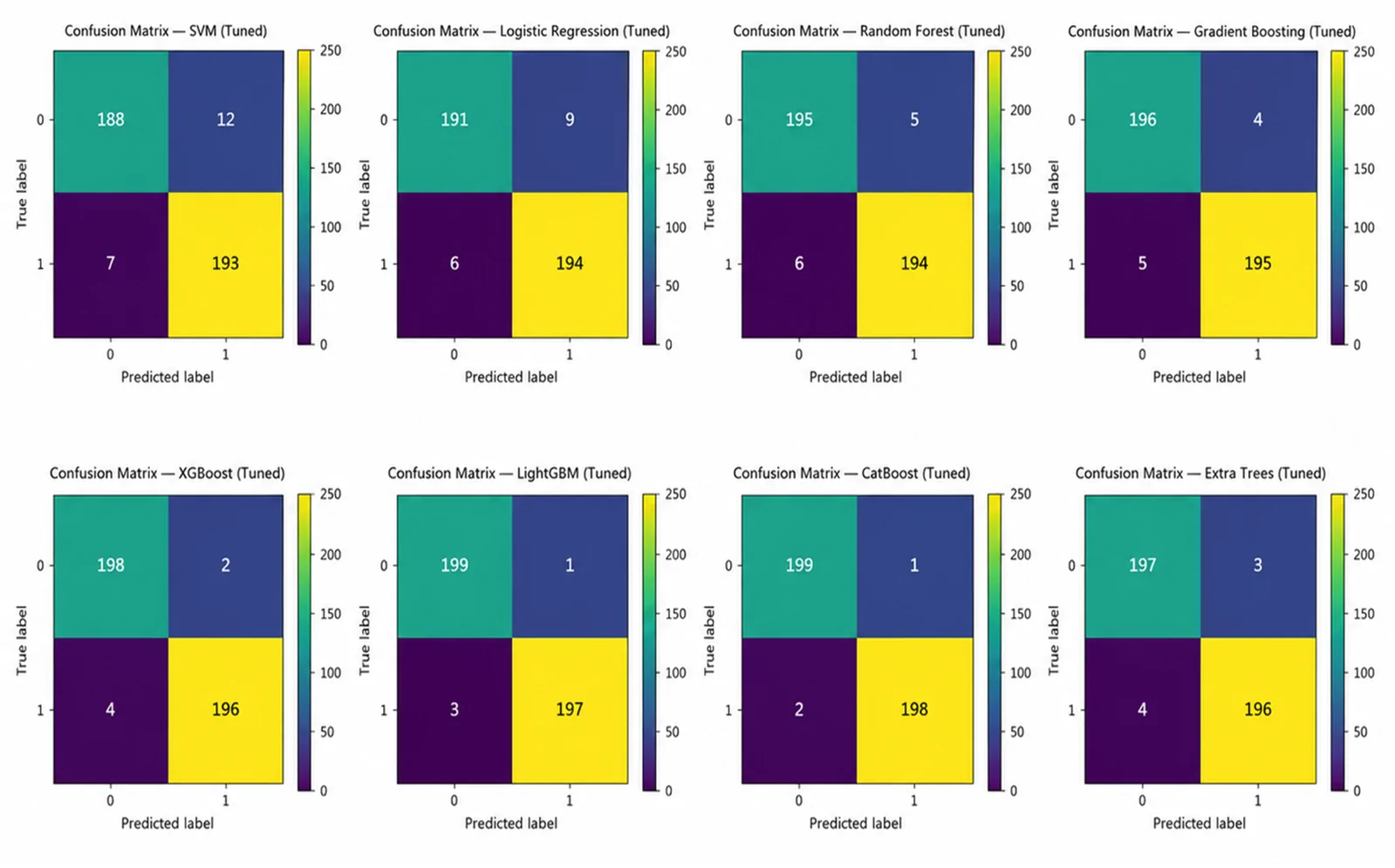
Confusion matrix of optimized models.

Twelve classification models was evaluated under the same repeated nested cross-validation framework. These included eight ML classifiers Logistic Regression, Support Vector Machine, Random Forest, Extra Trees, Gradient Boosting, XGBoost, LightGBM, and CatBoost and four neural network architectures DL1, DL2, DL3, and DL4. Hyperparameter optimization was conducted within the inner loop of the repeated nested stratified cross validation procedure, using ROC-AUC as the optimization criterion. The outer evaluation consisted of five stratified folds repeated five times, producing 25 held out test-fold evaluations for each model. Confusion matrices were generated for each model to analyse classification behaviour shown in Figure 6. The matrices summarize:

- True Positives (TP)
- True Negatives (TN)
- False Positives (FP)
- False Negatives (FN)

The evaluated models showed different trade-offs between sensitivity and specificity. Among all twelve models, CatBoost achieved the highest mean ROC-AUC of 0.909 ± 0.041, followed by Logistic Regression at 0.906 ± 0.041 and Random Forest at 0.905 ± 0.042. Gradient Boosting achieved a mean ROC-AUC of 0.903 ± 0.042 and therefore did not obtain the highest ROC-AUC. The differences between the leading models were small, and CatBoost was not statistically superior to Logistic Regression.

**Figure 7:**
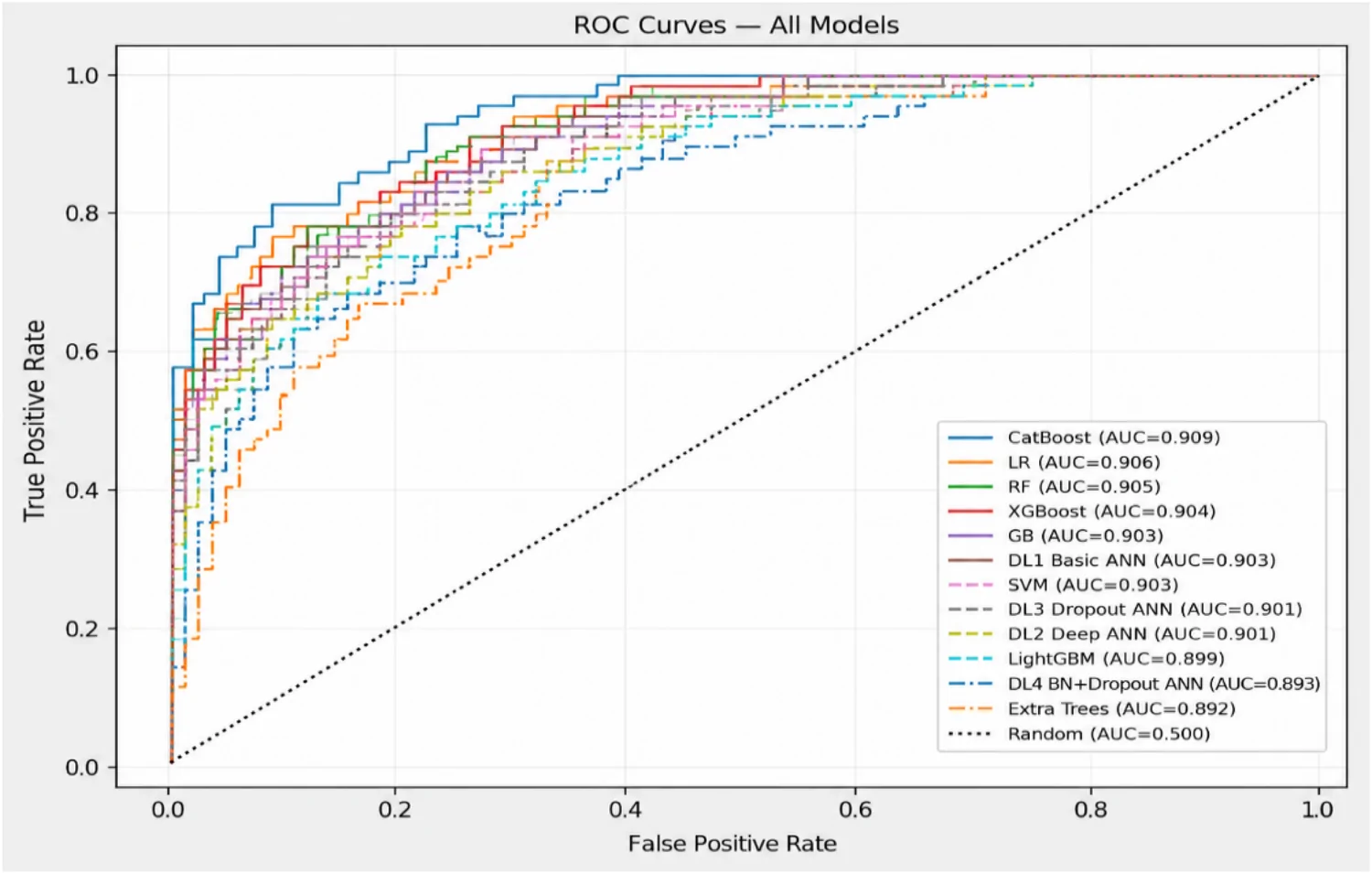
Combined ROC curves of ML and DL models.

As illustrated in Figure 7, the ROC curve plots the true positive rate against the false positive rate across varying thresholds. The area under the ROC curve (AUC) reflects overall classification capability independent of decision threshold. The combined ROC curves show that all evaluated models performed above the random baseline of AUC = 0.50. CatBoost achieved the highest mean ROC-AUC, followed closely by Logistic Regression and Random Forest. XGBoost, Gradient Boosting, DL1, and SVM also demonstrated competitive discrimination. However, the overlapping ROC-AUC values and statistical comparisons indicate that no single model was universally superior.

**Figure 8:**
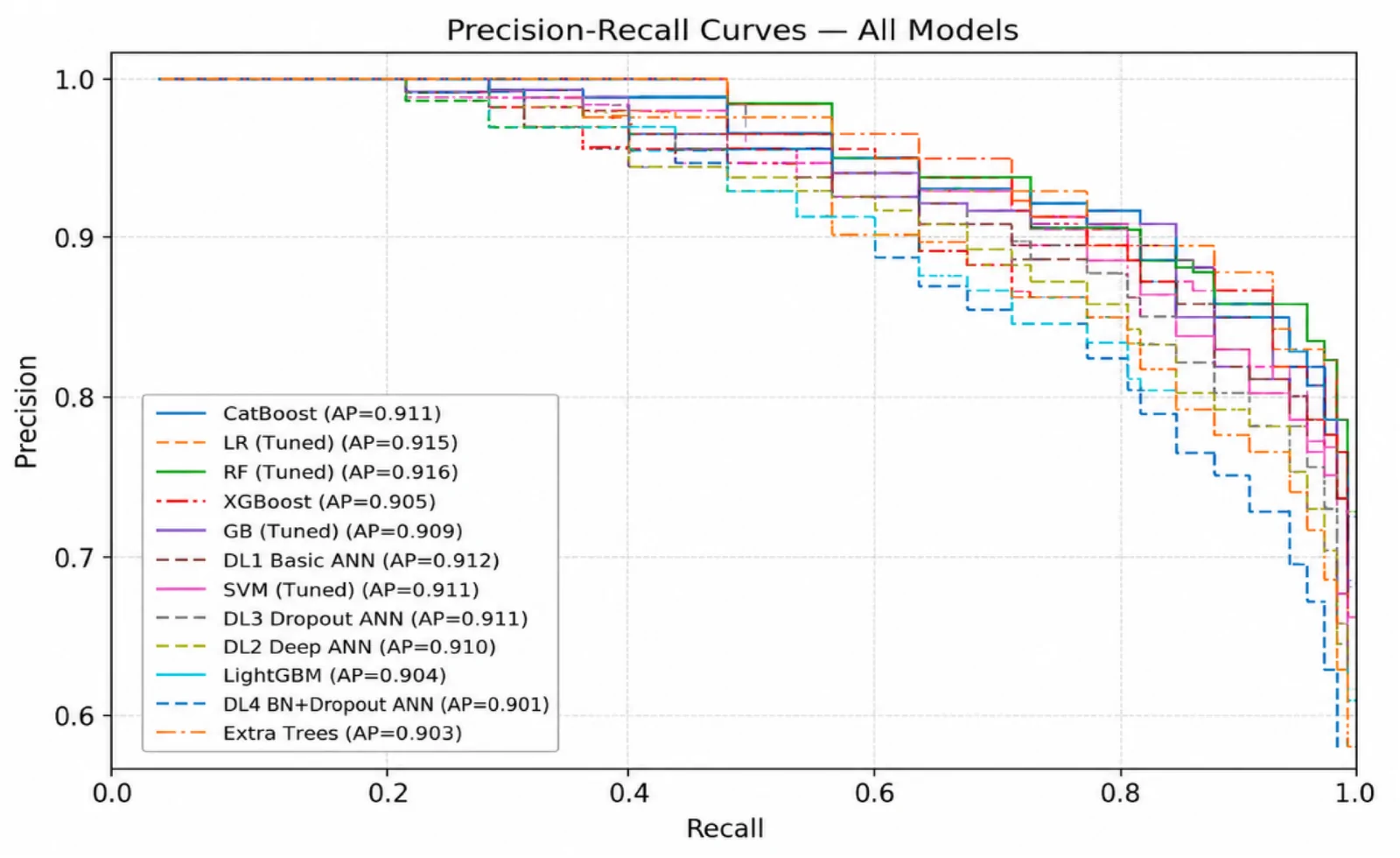
Precision, recall curves for all models.

As illustrated in Figure 8, precision, recall curves was generated for all twelve evaluated models to examine performance under varying recall thresholds. Random Forest achieved the highest mean PR-AUC of 0.916 ± 0.040, followed by Logistic Regression at 0.915 ± 0.041 and DL1 at 0.912 ± 0.045. Although several ensemble models performed strongly, the results did not demonstrate universal superiority of ensemble methods over the neural network architectures.

Four Artificial Neural Network architectures were implemented:

- DL1: Basic single hidden layer
- DL2: Multi-layer deep architecture
- DL3: Dropout-regularized ANN
- DL4: Batch Normalization + Dropout ANN

**Figure 9:**
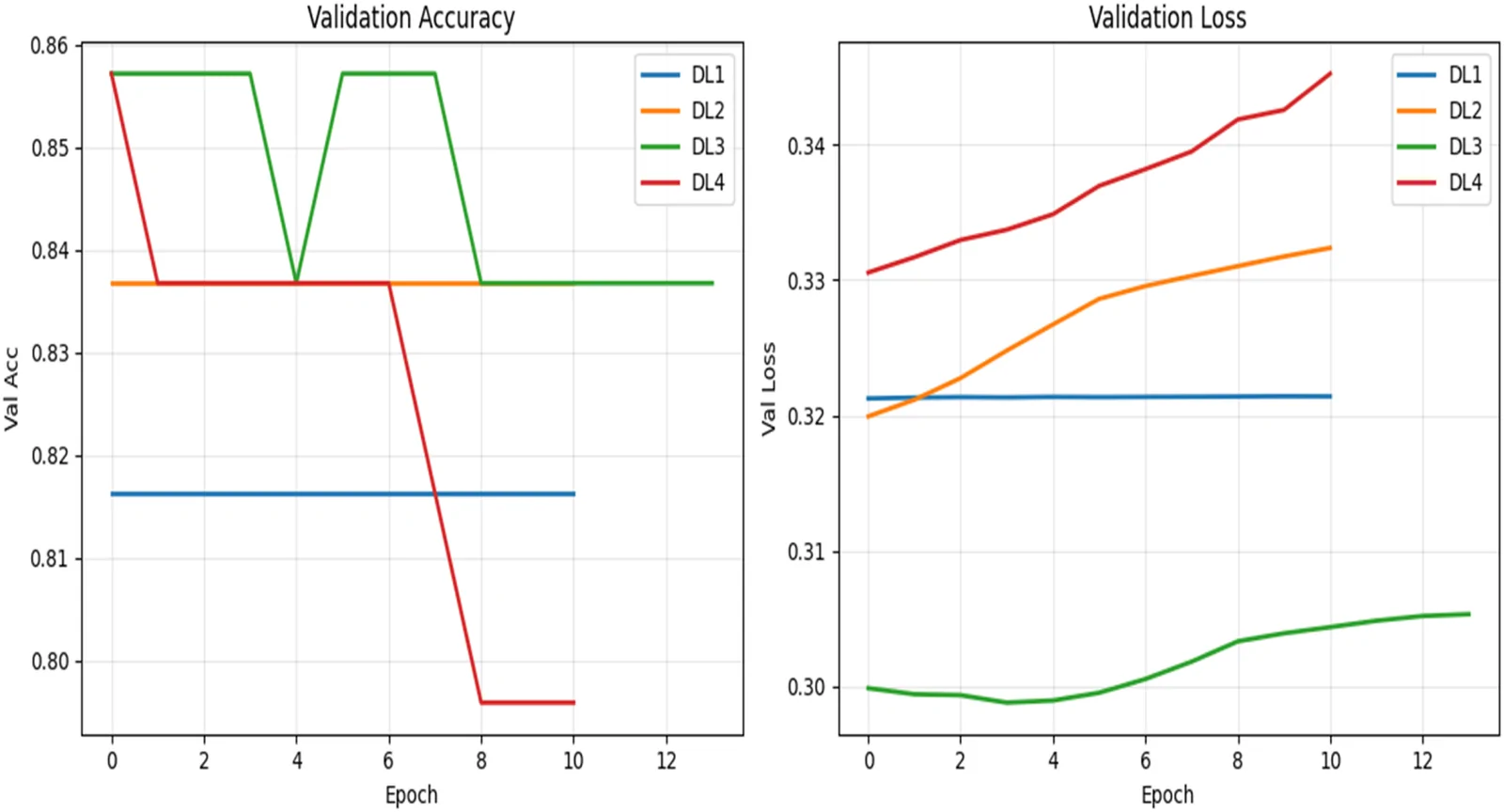
Validation accuracy and loss curves of deep learning models.

As illustrated in Figure 9, training and validation learning curves were plotted to analyse convergence behaviour. The basic ANN (DL1) achieved moderate predictive performance. Increasing network depth (DL2) improved training accuracy but showed minor overfitting tendencies. Incorporating dropout regularization (DL3) reduced validation fluctuation. The most stable performance was observed in DL4, where batch normalization improved convergence stability and reduced performance variance.

Table 2 summarizes across 25 outer test-fold evaluations, CatBoost achieved the highest mean ROC-AUC of 0.909 ± 0.041, followed by Logistic Regression at 0.906 ± 0.041 and Random Forest at 0.905 ± 0.042. CatBoost also achieved the highest mean accuracy of 0.844 ± 0.047 and F1-score of 0.862 ± 0.041. Random Forest achieved the highest mean PR-AUC of 0.916 ± 0.040. The differences between CatBoost and Logistic Regression were small, indicating that model ranking was metric-specific rather than evidence of universal superiority.

**Table 2:** Performance comparison of all models.

Model	Accuracy	Sensitivity	Specificity	F1	ROC-AUC	PR-AUC	Brier
CatBoost	0.844 ± 0.047	0.891	0.788	0.862 ± 0.041	0.909 ± 0.041	0.911 ± 0.045	0.124
Logistic Regression	0.843 ± 0.040	0.885	0.793	0.860 ± 0.034	0.906 ± 0.041	0.915 ± 0.041	0.124
Random Forest	0.826 ± 0.046	0.865	0.780	0.844 ± 0.040	0.905 ± 0.042	0.916 ± 0.040	0.128
XGBoost	0.835 ± 0.049	0.870	0.793	0.852 ± 0.043	0.904 ± 0.038	0.905 ± 0.045	0.130
Gradient Boosting	0.828 ± 0.046	0.878	0.770	0.848 ± 0.039	0.903 ± 0.042	0.909 ± 0.046	0.127
DL1	0.837 ± 0.042	0.879	0.786	0.854 ± 0.038	0.903 ± 0.043	0.912 ± 0.045	0.125
SVM	0.829 ± 0.042	0.880	0.768	0.849 ± 0.036	0.903 ± 0.041	0.911 ± 0.043	0.125
DL3	0.835 ± 0.043	0.879	0.783	0.853 ± 0.040	0.901 ± 0.044	0.911 ± 0.045	0.127
DL2	0.832 ± 0.039	0.875	0.780	0.849 ± 0.037	0.901 ± 0.044	0.910 ± 0.048	0.128
LightGBM	0.828 ± 0.054	0.867	0.781	0.846 ± 0.047	0.899 ± 0.042	0.904 ± 0.049	0.128
DL4	0.827 ± 0.047	0.859	0.788	0.843 ± 0.043	0.893 ± 0.047	0.901 ± 0.049	0.130
Extra Trees	0.811 ± 0.045	0.842	0.774	0.829 ± 0.041	0.892 ± 0.047	0.903 ± 0.043	0.135

The repeated evaluation changed the ranking observed in the original single 80:20 split. In particular, the batch-normalized dropout network, which previously obtained the highest single-split ROC-AUC, produced a mean ROC-AUC of 0.893 ± 0.047 under repeated evaluation. This finding demonstrates that rankings based on a single test partition can be unstable when only 303 observations are available.

- Statistical Comparison

The Friedman test identified overall differences among the evaluated models for ROC−AUC,χ2(11)=33.418,p<0.001; PR−AUC,χ2(11)=24.052,p=0.0125;andF1−score, χ2(11)=34.744,p<0.001. After Holm correction, CatBoost achieved significantly higher ROC-AUC than Extra Trees, DL4, and LightGBM. No pairwise PR-AUC comparison remained significant after multiple-comparison correction.

Importantly, CatBoost did not significantly outperform Logistic Regression in ROC-AUC, PR-AUC, or F1-score. McNemar's exact test also found no significant difference in their paired classification errors, p=0.804. Therefore, the results do not support a claim that CatBoost is universally superior to the simpler Logistic Regression model.

- Calibration Analysis

Model discrimination and calibration produced different rankings. Brier scores ranged from approximately 0.121–0.135. DL2 and DL3 produced calibration slopes closest to the ideal value of 1.0, whereas several tree and boosting models produced slopes greater than 1.0. CatBoost achieved the highest mean ROC-AUC but did not achieve the calibration slope closest to one. These findings demonstrate that a model with strong discrimination may not necessarily provide the most reliable probability estimates.

- Model Interpretation

Held-out permutation importance was calculated for CatBoost because it achieved the highest mean ROC-AUC. The number of major vessels produced the largest mean decrease in outer-fold ROC-AUC when permuted, 0.057 ± 0.030, followed by thalassemia status, 0.044 ± 0.023, and chest-pain category, 0.035 ± 0.025. ST depression, ST-segment slope, and sex made smaller contributions, while fasting blood sugar, cholesterol, and resting blood pressure showed little consistent importance.

- Limitations and Future Work

This study has several limitations. First, the dataset contains only 303 observations and does not provide patient identifiers, recruitment dates, hospital information or longitudinal outcomes. Second, the analysed file is a cleaned derivative with an already binary outcome and no missing values and therefore does not fully represent the data-quality challenges of the raw Cleveland dataset. Third, one pair of observations was identical, although removing one copy did not materially change the leading models' results. Fourth, repeated nested cross-validation provides stronger internal validation than a single train–test split but is not a substitute for independent external validation. Fifth, the fixed probability threshold of 0.50 was not selected using prospectively defined clinical costs. Finally, permutation importance and exploratory decision-curve analysis describe model behaviour within the current dataset and do not establish causal relationships or prospective clinical effectiveness. Future work should focus on validating the proposed models using larger and more diverse multicentre cardiovascular datasets to improve their generalizability and clinical applicability. In addition, prospective clinical evaluation, clinically informed threshold optimisation, and further investigations of model calibration, fairness across patient subgroups, and explainable artificial intelligence techniques are necessary before deployment in real-world clinical decision-support systems.

## 5 Conclusion

This study compared classical classifiers, modern tree ensembles, and regularized neural networks for heart disease classification using repeated nested internal validation. CatBoost produced the highest mean ROC-AUC, accuracy, and F1-score, while Random Forest achieved the highest PR-AUC. However, CatBoost did not significantly outperform Logistic Regression in discrimination, F1-score, or paired classification errors. These findings indicate that increasing model complexity did not guarantee a meaningful performance advantage on the small structured dataset. The revised analysis also demonstrated that model ranking depended strongly on the validation procedure. The batch-normalized dropout network that ranked first under the original single test split did not retain that position across repeated test folds. Model selection should therefore consider uncertainty, calibration, false-negative behaviour, interpretability, and computational complexity rather than relying on one maximum score. The study remains limited to internal validation using a cleaned 303-record benchmark dataset. The evaluated models are not ready for clinical deployment. Future work should include independent multicentre validation, prospective evaluation, clinically informed threshold selection, subgroup fairness assessment, and analysis of calibration and clinical net benefit under real-world distribution shift.

## Data Availability

The original contributions presented in the study are included in the article/Supplementary Material, further inquiries can be directed to the corresponding author.
